# Mitochondrial oncobioenergetic index: A potential biomarker to predict progression from indolent to aggressive prostate cancer

**DOI:** 10.18632/oncotarget.5487

**Published:** 2015-10-15

**Authors:** Praveen K. Vayalil, Aimee Landar

**Affiliations:** ^1^ Department of Pathology, Division of Molecular and Cellular Pathology, Center for Free Radical Biology, University of Alabama at Birmingham, Birmingham, Alabama, USA

**Keywords:** mitochondria, prostate cancer, oncobioenergetics, bioenergetics, mitochondrial oncobioenergetic index

## Abstract

Mitochondrial function is influenced by alterations in oncogenes and tumor suppressor genes and changes in the microenvironment occurring during tumorigenesis. Therefore, we hypothesized that mitochondrial function will be stably and dynamically altered at each stage of the prostate tumor development. We tested this hypothesis in RWPE-1 cells and its tumorigenic clones with progressive malignant characteristics (RWPE-1 < WPE-NA22 < WPE-NB14 < WPE-NB11 < WPE-NB26) using high-throughput respirometry. Our studies demonstrate that mitochondrial content do not change with increasing malignancy. In premalignant cells (WPE-NA22 and WPE-NB14), OXPHOS is elevated in presence of glucose or glutamine alone or in combination compared to RWPE-1 cells and decreases with increasing malignancy. Glutamine maintained higher OXPHOS than glucose and suggests that it may be an important substrate for the growth and proliferation of prostate epithelial cells. Glycolysis significantly increases with malignancy and follow a classical Warburg phenomenon. Fatty acid oxidation (FAO) is significantly lower in tumorigenic clones and invasive WPE-NB26 does not utilize FAO at all. In this paper, we introduce for the first time the mitochondrial oncobioenergetic index (MOBI), a mathematical representation of oncobioenergetic profile of a cancer cell, which increases significantly upon transformation into localized premalignant form and rapidly falls below the normal as they become aggressive in prostate tumorigenesis. We have validated this in five prostate cancer cell lines and MOBI appears to be not related to androgen dependence or mitochondrial content, but rather dependent on the stage of the cancer. Altogether, we propose that MOBI could be a potential biomarker to distinguish aggressive cancer from that of indolent disease.

## INTRODUCTION

Prostate cancer is a major cause of cancer-related mortality and morbidity in males especially in older men (60–70 years) worldwide [1]. Among American men, prostate cancer is the second leading cause of cancer death. The new estimated cases for prostate cancer in the United States for 2015 is about 220,800 and deaths from prostate cancer is about 27,540 [2]. The common use of predictors, such as preoperative serum prostate specific antigen (PSA) levels, has resulted in earlier diagnosis and intervention of prostate cancer [3]. However, a significant fraction of prostate cancers detected solely on the basis of an increased serum PSA are subclinical indolent tumors, while only a small fraction of these tumors actually progress into an aggressive form, providing a major clinical challenge to distinguish between the two. Accordingly, the future progress in combating prostate cancer will be highly dependent upon the availability of improved diagnostic tools to distinguish aggressive forms of cancer from indolent tumor early enough to deliver appropriate treatment strategies to achieve a disease-free state and to reduce over-diagnosis and over-treatment [4, 5].

Mitochondrial function, its genetics and energy metabolism have important roles in cancer development including tumor initiation and progression and are emerging indicators of prostate cancer biology [6]. In addition to their critical role in ATP production, mitochondria performs numerous other biochemical reactions and are the mediators of multiple cellular processes including catabolic and anabolic metabolism, signaling, generation of reactive oxygen species (ROS) and apoptosis [7]. Mitochondria are highly sensitive to stress and respond dynamically to the changes in their cellular microenvironment [8]. The macromolecules of the mitochondrion are susceptible to oxidative damage, which usually accompanies inflammation and other pathological stress. These changes can result in progressive shift in bioenergetic function and alterations in the metabolic phenotype as the disease progresses towards more severe clinical stages. Failure to remove damaged mitochondria by mitophagy and replace with healthy mitochondria has been suggested as one of the mechanisms for such changes [9–11]. Therefore, use of a “bioenergetic health index” as a new biomarker with prognostic and diagnostic value has been proposed recently [11].

During the multi-step process of carcinogenesis, mitochondrial function and bioenergetics of tumor cells (oncobioenergetics) are influenced by the alterations of oncogenes and tumor suppressor genes [12, 13]. Moreover, during the transformation of epithelial cells towards malignancy, interaction between cancer and different stromal cellular elements, including immune cells, may also modify the mitochondrial function of cancer cells. More importantly, unregulated proliferation of cancer cells and aberrant angiogenesis within the tumor mass eventually results in oxygen and nutrient deprivation. To survive in an ever-changing microenvironment within the tumor mass, cancer cell mitochondria have to functionally adapt to these changes [14]. Therefore, we hypothesized that mitochondrial oncobioenergetics of prostate cancer cells will be stably and progressively altered at each stage of the tumor development.

A number of studies have been carried out to determine the mitochondrial bioenergetics of well-established malignant prostate cancer cells lines widely used for prostate cancer research [15–19]. However, these cancer cell lines as well as the normal prostate epithelial cells are not genetically identical as they were obtained from different patients or healthy individuals. These cell lines are grown under tissue culture conditions for several passages in presence of antibiotics, which influence mitochondrial bioenergetic function [20, 21]. Moreover, most of the analyses were performed in isolated mitochondria or cells in suspension rather than intact adherent cells. In the present study, we have comprehensively evaluated the mitochondrial oncobioenergetics of a panel of adherent human syngeneic prostate epithelial cell lines with progressive malignant characteristics derived from RWPE-1 cells in real-time and validated this approach in other known prostate cancer cells of varying degree of malignancy.

To assess the mitochondrial oncobioenergetics, we used high-throughput respirometry, which allows simultaneous measurement of cellular OXPHOS and glycolysis in real-time in adherent cells grown in cell culture. Overall, our studies clearly demonstrate that under ambient atmospheric O_2_ there is a progressive change in the mitochondrial oncobioenergetic parameters as the prostrate epithelial cells gradually transform from indolent tumor to aggressive phenotype. We also show for the first time that mitochondrial oncobioenergetic index (MOBI), a mathematical representation of mitochondrial oncobioenergetic signature of a cancer cell, may be used to predict the aggressiveness of prostate cancer.

## RESULTS

### Mitochondrial content does not change with increasing malignancy

**Figure 1:**
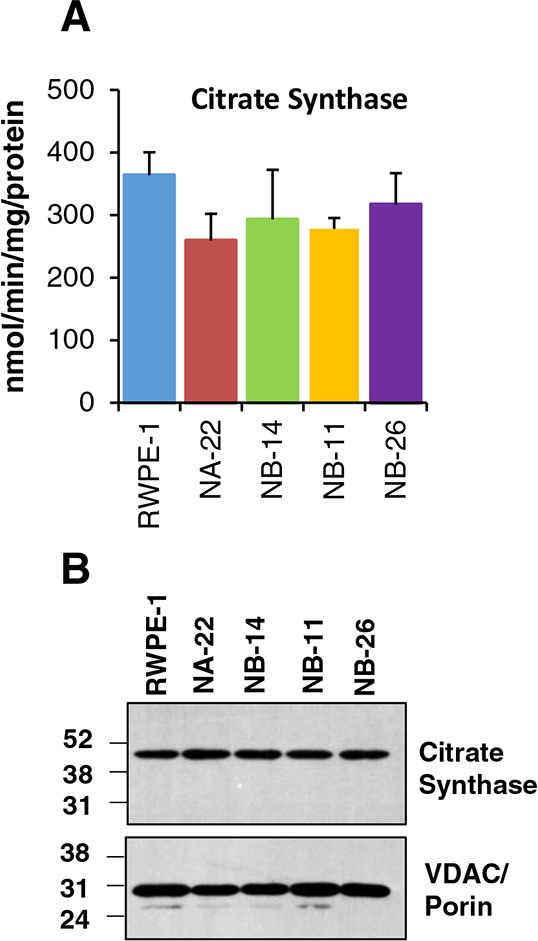
Mitochondrial content in RWPE-1 and it tumorigenic clones Citrate synthase enzyme activity **A.** Cells were lysed in a lysis buffer containing protease inhibitors as described in “Materials and Methods”. The data is represented as nmol/min/mg protein. The values are mean ± SE of two separate experiments performed in triplicates. Abundance of citrate synthase and VDAC/porin proteins in prostate epithelial cell lysate **B.** Total proteins isolated from cells were blotted with antibodies for CS and VDAC/porin. A representative image from two separate western blots is shown.

Dynamic changes in the mitochondrial oncobioenergetics of prostate epithelial cells during the transformation from early to late prostatic intra-epithelial neoplasia (PIN) and to invasive, metastatic prostate carcinoma was not systematically characterized. To evaluate the mitochondrial oncobioenergetics, RWPE-1 cells and its tumorigenic clones with increasing malignancy (RWPE-1 < WPE-NA22 < WPE-NB14 < WPE-NB11 < WPE-NB26) were used. Before attempting to measure the mitochondrial oncobioenergetics, we first determined the mitochondrial content of these cells. Studies have shown that measurement of citrate synthase activity is a good marker and correlate well with the mitochondrial content [22]. Our studies clearly demonstrate that citrate synthase activity does not change significantly in RWPE-1 or RWPE-1 - derived tumorigenic clones (Figure 1A). This is further validated at the protein level. Western blot analysis of total cell lysate and semi-quantification of the blots showed no difference in abundance of citrate synthase or VDAC protein among RWPE-1 and its tumorigenic clones (Figure 1B). This indicates that mitochondrial content of these cells does not change with tumorigenic transformation and thus any potential differences in oncobioenergetic parameters (see below) among these cells would not be influenced by the cellular mitochondrial content.

### OXPHOS decreases with increasing invasiveness of prostate cancer cells

To assess the mitochondrial oncobioenergetic parameters of RWPE-1 and its transformed clones comprehensively, oxygen consumption rate (OCR) was measured using high throughput respirometry. We performed a “mitochondrial stress test” (MiST) using modulators of mitochondrial function such as oligomycin, FCCP and antimycin A, which allows interrogation of certain components of mitochondrial oncobioenergetic function as described in “Materials and Methods”. Three assay media conditions were used: (*a*) standard assay medium which contained 5.0 mM glucose and 4.0 mM glutamine as energy substrates (*b*) substrate-restricted assay medium with glucose alone (5.0 mM), and *(c)* glutamine alone (4.0 mM).

**Figure 2:**
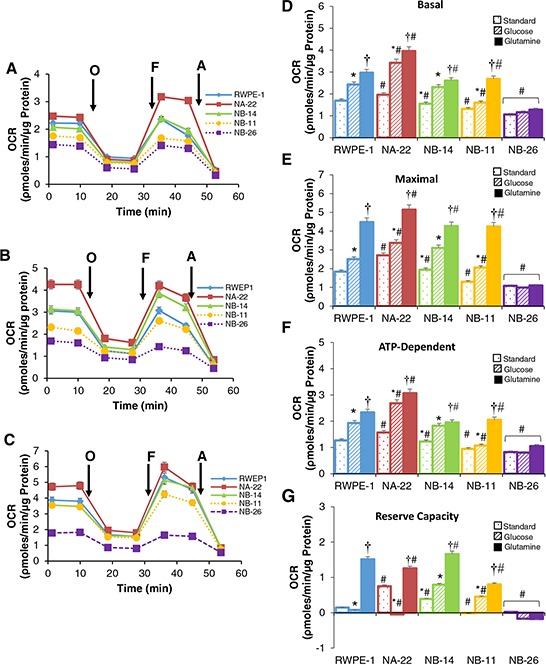
Oncobioenergetic profile of RWPE-1 and its clones analyzed by MiST in standard or substrate limited assay media Cells were plated on XF96 plates in standard XF assay media. The medium was removed and replaced with **A.** standard XF assay medium (DMEM, supplemented with 5.0 mM glucose and 4.0 mM glutamine without bicarbonate (pH 7.4)), or **B.** DMEM with glucose (5.0 mM) or **C.** glutamine (4 mM) as the only energy substrate and equilibrated 1 h before MiST. OCR of different cell lines was plotted against time after sequential injection of oligomycin (O); FCCP (F); and antimycin (A) Values are the mean ± SE of observations made from 15–30 wells from two separate experiments [♦RWPE-1; ■ WPE1-NA22; ▲ WPE1-NB14; ● WPE1-NB11; ■ WPE1-NB26]. Major oncobioenergetic parameters of RWPE-1 and it tumorigenic clones in presence of different energy sources **D–G.** Cells provided with standard (dotted) or in glucose (diagonal striped) or glutamine (solid filled) limited assay medium. Basal (D), maximum (E) ATP-dependent/oligomycin inhibitable (F) respiration and reserve capacity (G) were calculated from the OCR traces corresponding to each substrate as depicted in A-C. Values are the mean ± SE of observations made from 15–30 wells from two separate experiments. *p < 0.05 compared to standard assay media, † *p* < 0.05 compared to standard assay media and glucose restricted media; #*p* < 0.05 compared to RWPE-1 cells or WEP1-NA22 exposed to corresponding substrate.

Under standard assay media conditions, OCR trace normalized to protein concentration has clearly demonstrated a substantial modification of mitochondrial function among different clones when interrogated with modulators of mitochondrial function as shown in Figure 2A. Early pre-malignant cell line WPE1-NA22 showed an increase in basal (Figure 2D), maximal (Figure 2E), and ATP-dependent (Figure 2F) OCR compared to non-tumorigenic RWPE-1 cells or other tumorigenic clones. However, as the invasiveness increases (WPE1-NB11 and WPE1-NB26) these oncobioenergetic parameters are significantly reduced compared to RWPE-1 or WPE1-NA22. Late pre-malignant cell line WPE1-NB14 demonstrated an intermediate mitochondrial oncobioenergetic profile, which overlaps with that of RWPE-1 and significantly lower compared to WPE1-NA22. This suggests that as the pre-malignant cells progress towards an invasive phenotype, their mitochondrial function is also progressively diminished. Moreover, there is a significant inhibition of non-mitochondrial respiration as the cells become invasive (data not shown). However, no progressive changes were observed in proton leak with increasing malignancy among different cell lines (data not shown).

Most notable difference among the various mitochondrial oncobioenergetic parameters in these cell lines under standard assay media conditions is the reserve capacity. Reserve capacity is the difference between maximal and basal respiration, which is an estimate of the potential bioenergetic reserve the cell can call upon at times of stress [23, 24]. When cells are subjected to stress, mitochondrial reserve capacity is available to serve the increased energy demands for maintenance of organ function, cellular repair or detoxification of reactive species. Our study demonstrates that early and late pre-malignant non-invasive cells have a significantly higher reserve capacity compared to RWPE-1 cells (Figure 2G). On the other hand, early and late invasive cell lines (WEP1-NB11 and WEP1-NB26) totally lacked reserve capacity.

Next we tested the oncobioenergetic response of RWPE-1 cells and its tumorigenic clones to individual substrates, which was performed by pre-equilibrating for 1 hr. in an assay medium containing either glucose or glutamine alone and analyzed by MiST (Figure 2B and 2C respectively). The mitochondrial oncobioenergetic parameters of these cells in presence of glucose alone were significantly higher than in standard assay medium conditions. On the other hand, in the presence of glutamine alone as the energy source, basal (Figure 2D), maximal (Figure 2E), and ATP-dependent respiration (Figure 2F) and reserve capacity (Figure 2G) in RWPE-1, WPE-NA22, WPE-NB14 and WPE-NB11 cells were significantly higher compared to both standard assay media or in presence of glucose alone. It is interesting to note that the reserve capacity of WEP1-NA22, a cell line with high energy demand, is completely obliterated in presence of glucose alone and not in presence of glutamine (Figure 2G). Moreover, in presence of glutamine and glucose alone as substrate, the oncobioenergetic parameters increased rapidly as the RWPE-1 cells are transformed into WPE-NA22 and it diminished with increasing malignant phenotype. Remarkably, most invasive and malignant clone WPE1-NB26 (similar to DU145 cells) did not show any difference in their oncobioenergetic profile irrespective of the available substrate and was significantly lower compared to any other RWPE-1 tumorigenic cell lines.

### Metabolic phenotype shifts as RWPE-1 cells progress from indolent to invasive cancer cells

**Figure 3:**
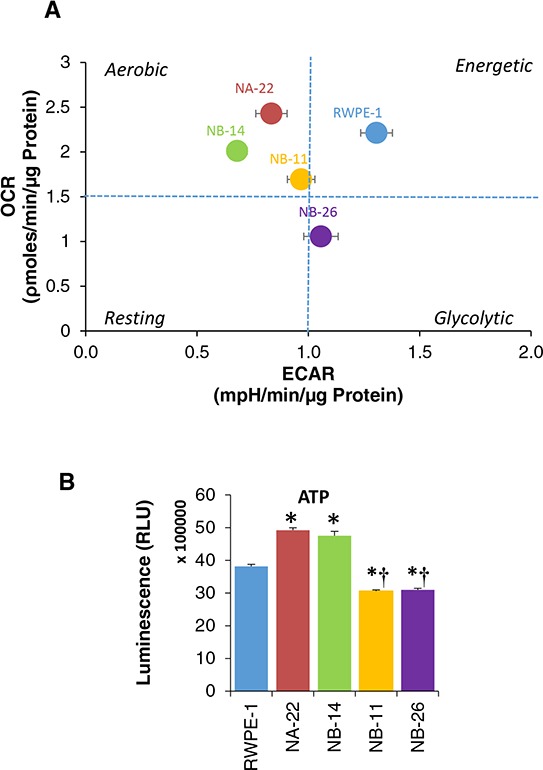
Metabolic phenotype and cellular ATP levels of RWPE-1 cells and its tumorigenic clones **A.** A representative XF PhenoGram by plotting basal OCR versus basal ECAR values obtained from the above experiment (Figure 2A) is shown. [●RWPE-1; ● WPE1-NA22; ● WPE1-NB14; ● WPE1-NB11; ● WPE1-NB26]. **B.** ATP levels in RWPE-1 and its tumorigenic clones. Cells were seeded at a density of 2 × 10^4^ cells/well in 96-well plates in triplicates in complete media as describe above and incubated overnight at 37°C, in 5% CO_2_. The ATP level was measured using CellTiter-Glo®.

An XF PhenoGram generated by plotting basal OCR vs. ECAR [14, 25], to describe the metabolic phenotype of each clone, demonstrated that RWPE-1 cells and its clones show a characteristic switch in their oncobioenergetics as their phenotype progresses from indolent to malignant tumor. Figure 3A shows that the five cell lines studied fall into three quadrants of the PhenoGram. RWPE-1 cells characteristically fall under energetic quadrant due to high OCR and ECAR and are have energetic phenotype. As they transform into pre-malignant (WPE1-NA22 and WPE1-NB14) and early invasive cells (WPE1-NB11) their energetics shifts to an aerobic phenotype (high OCR and low ECAR). However, invasive cells (WPE1-NB26) fall in the glycolytic quadrant as they have low OCR and high ECAR suggesting that prostate epithelial cells undergo a progressive shift in their metabolic phenotype during the multi-step process of prostate carcinogenesis.

### Total cellular ATP levels are reduced with invasive phenotype

The above results suggest that shift in metabolic phenotype of the cells as they transform from pre-malignant cells into highly invasive cancer cells, may be reflected at cellular ATP levels. Therefore, next we asked whether the oncobioenergetic status of the cells reveal the total cellular ATP levels in RWPE-1 and its tumorigenic clones. Same number of cells from each clone was analyzed by a chemi-luminescence method for ATP levels (Figure 3B). Both early and late pre-malignant cells (WPE1-NA22 WPE1-NB14) had significantly higher levels of ATP compared to RWPE-1 cells. Although cellular ATP levels were significantly lower in cells with invasive phenotype (WPE1-NB11 and WPE1-NB26) compared to non-tumorigenic and premalignant cells, they produced a considerable amount of ATP (80% of RWPE-1) even though the cell's respiration is very low compared to other clones.

### FAO decreases with malignancy

**Figure 4:**
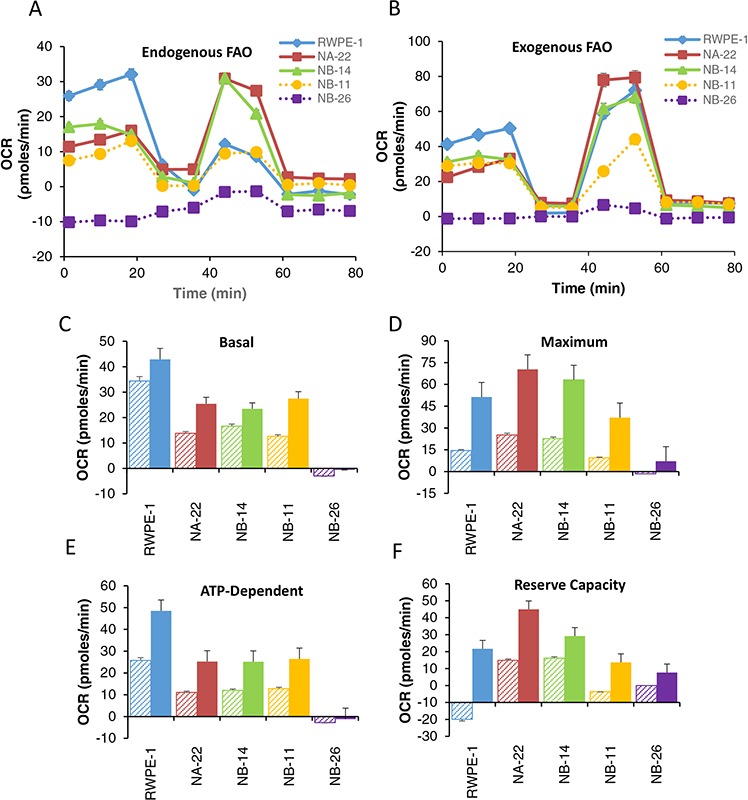
Endogenous and Exogenous FAO in RWPE-1 and its tumorigenic clones Cells were seeded and the growth medium was then replaced next day with DMEM containing reduced concentrations of glucose, and glutamine, to deplete endogenous substrates within the cell. Endogenous FAO is performed in KHB containing BSA. For exogenous FAO, instead of BSA, Palmitate-BSA was added as the only energy source. OCR due to FAO was determined by treating the cells with ETO (40 μM) for 15 min prior to the addition of BSA and MiST was performed as described in “Materials and Methods”. **A and B.** OCR trace due to endogenous and exogenous FAO [♦RWPE-1; ■ WPE1-NA22; ▲ WPE1-NB14; ● WPE1-NB11; ■ WPE1-NB26]; **C.** Basal, **D.** Maximum, **E.** ATP or oligomycin dependent respiration due to FAO and **F.** reserve capacity. Diagonally stripped bars represent endogenous and solid bars represent exogenous FAO. Values are the mean ± SE of observations made from 8–16 wells in two separate experiments. *p < 0.05 compared to RWPE-1, † *p* < 0.05 compared to WEP1-NA22.

Fatty acids constitute a major source of cellular energy for cancer cells. Earlier studies support the hypothesis that FAO is a dominant oncobioenergetic pathway in prostate cancer [26]. However, no direct evidence is available to support the notion that FAO changes with malignancy of the tumor. Therefore, we set forth to comprehensively determine the endogenous and exogenous FAO in RWPE-1 cells and it tumorigenic clones. All RWPE-1 tumorigenic clones except highly invasive WPE1-NB26 could oxidize endogenous and exogenous fatty acids as a source of energy (Figure 4A & 4B). In non-tumorigenic RWPE-1 cells, basal OCR (Figure 4C) and ATP-dependent OCR (Figure 4E), either endogenous or exogenous, were significantly higher compared to tumorigenic cells. On the other hand, early (WPE1-NA22) and late (WPE1-NB11) pre-malignant cells showed increased reserve capacity (Figure 4F) due to elevated maximal respiration (Figure 4D) irrespective of the source of fatty acids. It is interesting to note that reserve capacity of RWPE-1 cells due to endogenous FAO is significantly lower due to reduced maximal respiration. This may be due to substrate depletion as the basal respiration of RWPE-1 is twice more than the other cell lines. FAO-dependent maximal (Figure 4D) and reserve capacity (Figure 4F) decreases progressively with malignant phenotype from premalignant (WPE1-NA22) to invasive (WPE1-NB26) prostate cancer cells. Although, basal (Figure 4C) and ATP-dependent OCR (Figure 4E) is lower than non-tumorigenic cells, it does not decrease progressively with increasing malignancy. Surprisingly, highly invasive cancer cell clone, WPE1-NB26, did not utilize either endogenous or exogenous palmitate at all.

### Glycolytic rate is elevated with malignancy

**Figure 5:**
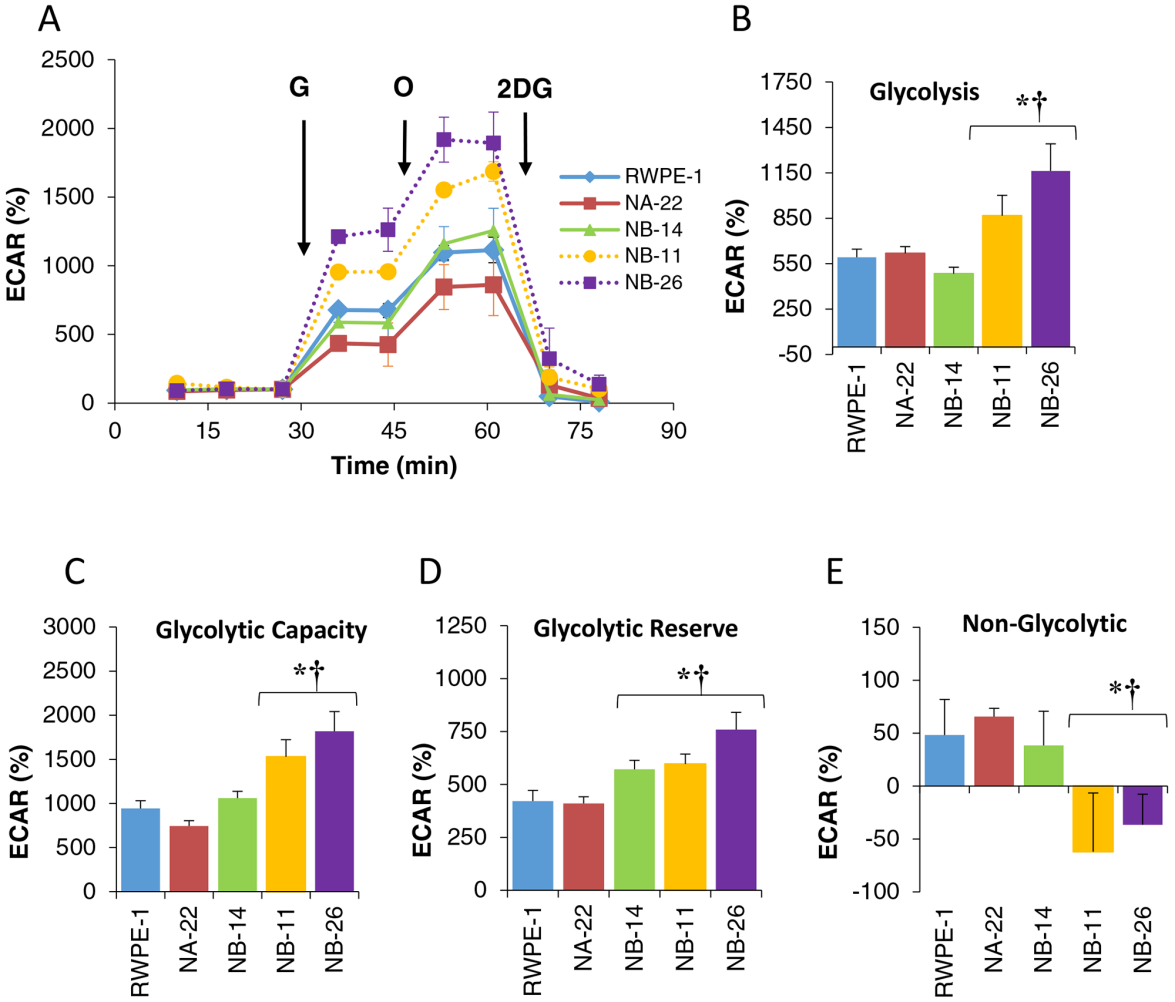
Glycolytic profile of RWPE-1 and its clones analyzed by GlyST Cells were plated on XF96 plates in complete media. After 24 hr. the media was changed to an assay medium containing 2 mM glutamine without glucose for 1.0 hr. After three ECAR measurements glucose (10 mM final) Oligomycin (1.0 μM final) and 2DG (100 mM final) were added sequentially. The resulting ECAR trace was normalized to the baseline (ECAR without glucose) represented in percent and was used to calculate various glycolytic parameters of each cell as described in the “Materials and Methods”. The values are mean ± SE of 12 to 30 wells from two separate experiments. *p < 0.05 compared to RWPE-1 and † compared to WPE1-NA22.

Cancer cells are characterized by their glycolytic phenotype even in the presence of ample amount of oxygen (O_2_), a phenomenon known as Warburg effect. Earlier studies using complex tissue preparations (human prostate and prostatic adenoma) and rat ventral prostate cells it was reported to exhibit high aerobic glycolysis [19]. However, comprehensive analysis of glycolytic parameters using human prostate epithelial cells and other tumorigenic cells with different stages of malignancy are still elusive. To analyze the glycolytic profile in detail, RWPE-1 and its tumorigenic cells lines were subjected to GlyST. By GlyST, four major glycolytic parameters could be analyzed from the ECAR trace as shown in the Figure 5A. When normalized to baseline (ECAR in the absence of glucose and represented as percentage), glycolysis (Figure 5B), glycolytic capacity (Figure 5C) and glycolytic reserve (Figure 5D) increased significantly (1.5–2 fold) as soon as the cells achieved an invasive (WPE1-NB11) and metastatic phenotype (WPE1-NB26). Glycolytic parameters, on the other hand, did not change drastically among non-tumorigenic cells (RWPE-1) and early (WPE1-NB22) and late (WPE1-NB14) pre-malignant cells. Moreover, early premalignant cells have higher non-glycolytic acidification compared to invasive cells (Figure 5E).

### Mitochondrial oncobioenergetic index predicts prostate cancer aggressiveness

**Figure 6:**
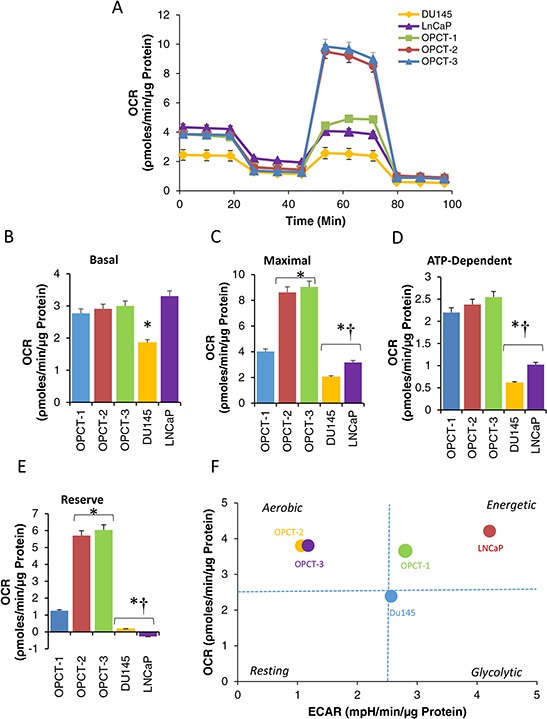
Oncobioenergetic profile of prostate cancer cell lines of varying degree of malignancy developed from different patients Cells were plated on XF96 plates in standard XF assay media. The medium was removed and replaced with standard XF assay medium (DMEM, supplemented with 5.0 mM glucose and 4.0 mM glutamine without bicarbonate (pH 7.4)) and equilibrated 1 h before MiST. OCR of different cell lines was plotted against time after sequential injection of oligomycin (O); FCCP (F); and antimycin **A.** [♦OPCT-3; ■ OPCT-2; ▲ OPCT-1; ● DU145; ■ LNCaP]. Major oncobioenergetic parameters of RWPE-1 and it tumorigenic clones in presence of different energy sources **B–E.** Basal (B), maximum (C) ATP-dependent/oligomycin inhibitable (D) respiration and reserve capacity (E) were calculated from the OCR traces corresponding to each substrate as depicted in Figure 6A. Values are the mean ± SE of observations made from 15–30 wells from two separate experiments. *p < 0.05 compared to standard assay media, † *p* < 0.05 compared to standard assay media and glucose restricted media; #*p* < 0.05 compared to RWPE-1 cells or WEP1-NA22 exposed to corresponding substrate. Metabolic phenoptype of prostate cancer cell lines of varying degree of malignancy developed from different patients is depicted as a representative phenogram **F.**

Once we comprehensively analyzed the oncobioenergetics of RWPE-1 cells and its tumorigenic clones, we next sought to determine whether these changes follow in other prostate cancer cell lines of various stages of malignancy both obtained from the primary site as well as distant metastatic sites. Three cell lines (OPCT-1, −2 and −3) isolated from localized prostate cancer of stage T1c, T2a and T3a with Gleason score ≥6 respectively and well established metastatic cell lines, DU145 (brain) and LNCaP (lymph node) were studied. MiST was performed using these cell lines in standard assay media conditions (Figure 6A). Our studies demonstrate that all cells, except DU145, showed higher basal respiration (Figure 6B). Maximal (Figure 6C) ATP-dependent respiration (Figure 6D) and reserve capacity (Figure 6E) were significantly lower in metastatic cells. However, these parameters were significantly elevated in OPCT-1, −2, −3 cells. Phenogram generated for these cell lines demonstrates that OPCT-1 cells behaved much like a highly energetic cell line and OPCT-2, and −3 were aerobic phenotype (Figure 6F). Unlike WEP1-NB26 cells, LNCaP and DU145 malignant cells were either energetic or slightly glycolytic phenotype respectively (Figure 6F). This suggests that tumor cells of varying degree of malignancy have overlapping mitochondrial oncobioenergetic and metabolic phenotypes, including reserve capacity (Figure 2G) and individually each parameter does not correlate with the degree of malignancy.

**Figure 7:**
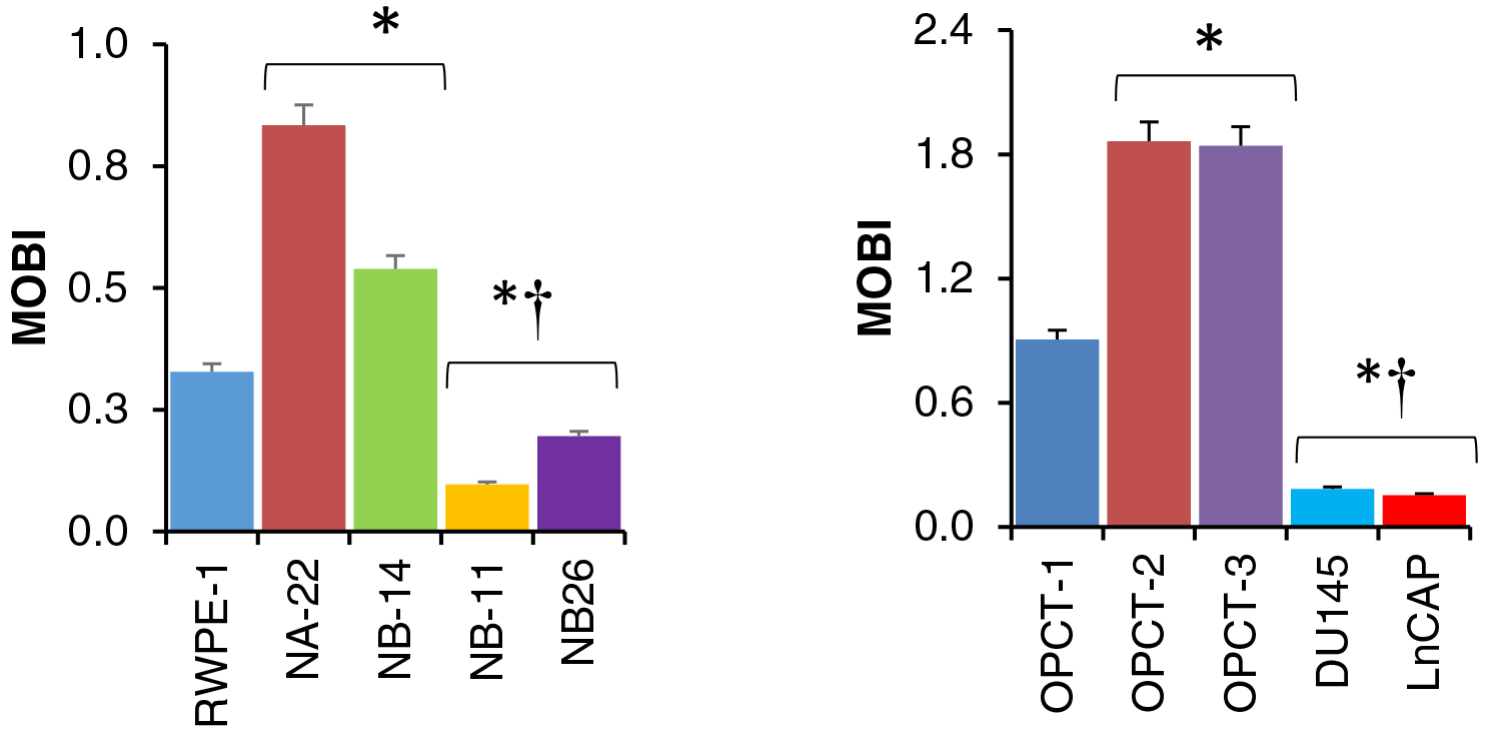
Mitochondrial Oncobioenergetic index of prostate epithelial cells MOBI of RWPE-1 and its tumorigenic clones **A.** and few commercially available prostate cancer cell lines of various degrees of malignancy **B.** was calculated from the oncobioenergetic parameters obtained from OCR trace in Figure 2 and Figure 6 respectively as described in the “Materials and Methods”.

To resolve this problem, we mathematically expressed the mitochondrial oncobioenergetic function utilizing mitochondrial oncobioenergetic index (MOBI) as a measure. We used a modified equation as described in “Materials and Methods” [11]. Our study demonstrates that MOBI, under standard assay conditions, has a strong relationship with the malignant phenotype of the cells (Figure 7A). We demonstrate for the first time that the MOBI increases as the prostate epithelial cells transform into localized pre-malignant stages of tumor (WPE1-NA22 and WPE1-NB14) compared to the normal clone (RWPE-1), while, it drops below the normal as soon as they become aggressive or malignant (WEP1-NB11 and WEP1-NB26). This observation was further tested in five commercially available prostate cancer cell lines of different stages of tumor growth obtained from primary tumor and metastatic site (Figure 7B). Localized benign OPCT-1 cells demonstrated a slight increase in MOBI compared to RWPE-1 cells. On the other hand, OPCT-2 and OPCT-3 showed a characteristic increase in MOBI as demonstrated for the non-invasive cells such as WEP1-NA22 and NB14. Highly malignant or metastatic cells, obtained from secondary sites (DU145 from brain, LNCaP from lymph node) demonstrated a very low MOBI similar to WPE1-NB11 and NB26). Moreover, MOBI seems to be independent of androgen sensitivity and mitochondrial content, as the cells used to validate our hypothesis had different mitochondrial content [15], and is more related to their malignant stage.

## DISCUSSION

Several studies have shown alterations in the proteins of mitochondrial electron transport chain, especially-F1-ATPase and HSP60, in a number of cancers [27–30] that could in turn affect the mitochondrial oncobioenergetic functional parameters. The ratio of β-F1-ATPase to HSP60 (mitochondrial) relative to GAPDH (glycolytic potential) has been shown to be associated with patient survival [28] suggesting that mitochondrial oncobioenergetic functional profile may be a useful parameter to predict the aggressiveness of cancer. Regardless of the specific mechanism by which they contribute to mitochondrial dysfunction, the oncobioenergetic functional signature of epithelial cells during the step-wise progression from normal to aggressive cancer has not been explored in prostate cancer. Therefore, in the present study, we focused on elucidating in real-time the mitochondrial oncobioenergetic signature of five prostate epithelial cells of same genetic background and other cells of different stages of malignancy ranging from normal to highly invasive phenotype. Elucidating the characteristic mitochondrial oncobioenergetic signature of cancer cells at each stage of tumor development may not only predict aggressive prostate cancer from the benign tumor but also could unveil previously unidentified molecular targets that would assist in the development of new treatment strategies for prostate cancer sparing normal cells. In this study, we provide the evidence that alterations in mitochondrial oncobioenergetic signature is a characteristic feature of prostate epithelial cells as they progress from benign to malignant phenotype and establish a quantitative approach that could be utilized in identifying the aggressive phenotype of the tumors.

In the present study, we used RWPE-1 cells and its tumorigenic clones. RWPE-1 cells were developed by immortalizing non-neoplastic epithelial cells with a single copy of human papilloma virus (HPV) −18 DNA. The tumorigenic clones were developed by exposing RWPE1 cells to a potent carcinogen *N*-Methyl- *N*-nitrosourea (MNU) and sequentially selecting *in vitro* and *in vivo* based on their malignant potential [31]. These tumorigenic cell lines are well suited to study the stepwise changes in the mitochondrial oncobioenergetics occurring during the multi-step process of prostate tumorigenesis. Because, RWPE-1 is derived from the peripheral zone which comprises about 70% of the gland and is the major functional component of the human prostate gland. The peripheral zone is the major region of the gland where majority of the common malignant cancers (70–80%) originate in humans. As these tumorigenic clones were derived from a single parent cell line, they all have common genetic background and mimic different stages of prostate cancer and progression to invasive cancer [32, 33]. WPE1-NA22 clone is the least invasive and tumorigenic, the WPE1-NB14 intermediate and WPE1-NB11 and WPE1-NB26 is highly invasive and tumorigenic, which is comparable to the widely used malignant prostate cancer cell line DU145 [31]. Moreover, unlike other published studies [15–19], all the cell lines are grown under same growth or culture conditions in the absence of antibiotics and reduced serum concentrations (2%) if used and measured in real-time in cultured adherent cells.

The overall activity of oxidative phosphorylation in the cell is the result of both bioenergetic competence of the organelles and the mitochondrial content [28]. The mitochondrial content in the cell is regulated both during development and by cell type-specific programs [34]. Therefore, it is possible that the alterations in mitochondrial content may occur during multi-step tumor development and should be ruled out prior to the assessment of mitochondrial oncobioenergetics. Our study has clearly demonstrated that mitochondrial content of these cells do not vary drastically by tumorigenic transformation. A previous study has also indicated that the abundance and activity of mitochondrial proteins, such as β-F_1_-ATPase and HSP60, in normal and malignant prostate biopsies are similar [30]. Therefore, any change in the mitochondrial oncobioenergetic profiles or mitochondrial dysfunction in the tumorigenic prostate cell lines from the normal counterpart may be due to the differences in the bioenergetic competence of the organelle.

Prostate cancer tissue have been shown to possess a distinct metabolic signature compared to benign prostate tissue, indicating that there are consistent metabolic changes occur within the prostate upon transformation [5, 35, 36]. These metabolic changes should reflect in the mitochondrial oncobioenergetic functional profile, because mitochondria occupy a crucial position at the center of all the metabolic pathways and they also respond dynamically to the changes in the microenvironment [11]. In order to take a fresh look at altered mitochondrial oncobioenergetic profiles by employing high-throughput respirometry, we determined if any differences are observable between cancer, premalignant (early and late) and normal cells originated from the same parent cell line RWPE-1 [31–33]. Our study clearly demonstrated that OXPHOS is dramatically elevated as soon as the normal cells transform into premalignant cells and then drops as the cells develop aggressive prostate cancer phenotype. Concurrently, glycolysis rapidly declines as the cells are transformed to pre-malignant cells compared to non-tumorigenic cell line and is elevated as the cells become malignant. As a result, bioenergetically, each cell line could be demarcated into energetic (RWPE-1), aerobic (WPE1-NA22, WPE1-NB14 and WPE1-NB11) and glycolytic phenotype (WPE1-NB26). Overall, it suggests that highly malignant prostate epithelial cells follow a classical Warburg phenomenon.

Oncobioenergetic profile of prostate cancer cells cannot be universally applied to all cancer types and each type of tumor may have its own characteristic oncobioenergetic profile For example, studies have shown that glioma cells exhibit an increased OXPHOS as the glioma cells become more invasive and chemo-resistant [37]. Further detailed characterization of the oncobioenergetic profile of cancers of each organ should be performed in detail to establish this idea.

Unlike other normal mammalian cells, prostate epithelial cells secrete enormous amounts of citrate into the prostatic fluid and require a special intermediary metabolism. Prostate epithelial cells are thought to derive energy via aerobic glycolysis with less dependence on aerobic oxidation [19, 38]. Our studies show that to meet the precursor demands for the continued synthesis of citrate and provide ATP to meet the energy demands, prostate cells utilize not only glucose via glycolysis but also utilize glutamine efficiently. Although the results are not direct proof for the substrate preferences of these cells, it provides an indirect evidence for the potential metabolic pathways utilized by these cells. We showed that glutamine elevated all the oncobioenergetic parameters in these cells compared to glucose alone. These differences in the oncobioenergetic profile in presence of different substrates are attributable to the ATP demand. When glucose is the sole substrate, cells could generate ATP both by glycolysis as well as Krebs cycle. However, glutamine increases ATP demand, as it does not support glycolysis, which result in increased basal, ATP-dependent and uncoupled respiration to meet the energy demands. It is also interesting to note that in presence of glucose, the reserve capacity of high energy demanding WEP1-NA22 cells is completely obliterated (both basal and maximal respiration are the same) but, significantly elevated in presence of glutamine. Accumulation of citrate in prostate cells is mediated by the competitive inhibition of mitochondrial *cis*-aconitase by Zn^2+^ ions under physiological conditions, which block the conversion of citrate to aconitate [17–19, 38]. Therefore, the possible explanation for the reduced uncoupled respiration in presence of glucose may be due to truncated Krebs cycle resulting from the partial inhibition of aconitase enzyme, which is reversed by the presence of glutamine in the culture medium. Thus, we hypothesize that glutamine could be a good source of energy production through Krebs cycle and at the same time a precursor for citrate synthesis through reductive carboxylation [39, 40] depending on the requirements in normal prostate epithelial cells and cells at various stages of the tumor progression. To conclude, it is more important to note that as the cells gradually acquire a more invasive phenotype; OXPHOS is progressively reduced irrespective of the substrate utilized for energy production. Therefore, use of modulators of metabolism, such as dichloroacetate (DCA; an inhibitor of pyruvate dehydrogenase kinase), 2DG (inhibitor of hexokinase), or metformin (inhibitor of mitochondrial complex I) [41] as a therapeutic approach to prostate cancer would be justified [42].

Prostate epithelial cells have been shown to uptake fatty acids (palmitic acid) over glucose [43]. Gene expression analysis showed that in more than 50% of the prostate cancer patient samples CPT1B enzyme mRNA, a rate limiting enzyme involved in the β-oxidation of FA, was elevated [44] and proposed that FAO is a prominent oncobioenergetic pathway in prostate cancer [26]. However, there is no direct evidence to show the extent to which the prostate epithelial cells utilize fatty acids for β-oxidation depending on the aggressiveness. Our study demonstrates that both endogenous and exogenous FAO is significantly lower in early and late premalignant and early invasive cells compared to RWPE-1. More interestingly, utilization of exogenous palmitate for ATP synthesis is negligible in highly invasive cells. Therefore, it is likely that in invasive cancer cells, most of the fatty acid uptake may be directed for lipid synthesis; however, this needs further investigation. The lower energy requirement of metastatic cells may be met through glycolysis and Krebs cycle utilizing glucose and/or glutamine. It is also possible that metastatic prostate cancer cells may have other substrate preferences, such as lactate [45, 46] and may directly contribute to the mitochondrial oncobioenergetics. However, further investigations are required to establish this view.

One of the major clinical problems in newly diagnosed men with prostate cancer is to distinguish aggressive prostate cancer from indolent disease. As an attempt to use mitochondrial oncobioenergetics as a potential marker for tumor aggressiveness, we introduce in this manuscript the concept of MOBI in prostate cancer. We hypothesized that as the normal cell transforms into a pre-malignant cell and progresses through different stages into aggressive tumor, oncobioenergetics at each stage will also change correspondingly to support the growth of tumor cells. The results reported in this work provides for the first time a mathematical analysis of mitochondrial oncobioenergetic profile of cancer cells that could be related to the aggressiveness of prostate cancer. We demonstrate here that as the normal cells transform into localized premalignant tumor, they tend to have a very high MOBI than the normal cells and as the cells achieve invasive properties, the MOBI falls far below the MOBI of normal cells. Changes in MOBI occur irrespective of their differences in the individual oncobioenergetic parameters or androgen requirement. Although, high-throughput respirometry is not a feasible approach to determine the mitochondrial function *in vivo*, certain novel imaging techniques to determine various parameters of mitochondrial function [47] would be a novel approach for translational studies to distinguish aggressive prostate cancer. In conclusion, these results demonstrate for the first time that mitochondrial oncobioenergetic signature of prostate epithelial cells stably and gradually changes at each stage of the tumor progression and MOBI could be a potential biomarker to distinguish the aggressive phenotype from indolent one.

## MATERIALS AND METHODS

### Cell lines and cell culture methods

RWPE-1 (CRL-11609), WPE1-NA22 (CRL-2849), WPE1-NB14 (CRL-2850), WPE1-NB11 (CRL-2851) and WPE1-NB26 (CRL-2852) were purchased from American Type Culture Collection (ATCC) Manassas, VA. All the cells were grown in Keratinocyte Serum Free Medium (K-SFM) obtained from GIBCO, Life Technologies (Cat# 17005-042) supplemented with growth factors (0.05 mg/mL bovine pituitary extract, 5 ng/mL epidermal growth factor) at 37°C, in 5% CO_2_ without any antibiotics. Medium was replaced three times a week with fresh K-SFM and passaged every five days in T-75 flask.

DU145, and LNCaP cells were a gift from Dr. Buchsbaum, Professor and Director of Radiation Oncology, UAB and were originally obtained from ATCC (Manassas, VA). OPCT1, 2 and 3 prostate tumor cell lines were obtained from Asterand (Detroit, MI) and all these cell lines were regularly cultured and sub-cultured in K-SFM media supplemented with EFG, bovine pituitary extract at low FBS (2%) concentration without any antibiotics. Cells were plated in specialized Seahorse culture plates in the same growth medium without serum to minimize the effect of serum on the bioenergetic function, 24 h before the start of the assay as described in below in “Materials and Methods section”.

### Oncobioenergetic measurements

Extracellular flux (XF) analyzer (Seahorse, Billerica, MA) was used to measure the mitochondrial oncobioenergetics. XF analyzer simultaneously measures in real-time the two major energy yielding pathways-cellular aerobic respiration and glycolysis. It monitors O_2_ consumption rate (OCR; due to mitochondrial respiration) and extracellular acidification rate (ECAR; predominantly due to glycolysis) in real-time [24, 48, 49]. All the cells are subjected to three bioenergetic tests - mitochondrial and glycolytic stress test (MiST and GlyST) and ability to oxidize fatty acids (FAO). Cells were plated at an optimal cell density which was determined for each cell line before the analysis and found to be 3 × 10^4^ cells/well for XF^e^96 analyzer. Cellular response to mitochondrial stressors were performed by pre-treating the cells with 4-hydroxynonenal (4-HNE; 50 μM) an hour before the XF analysis.

#### MiST

After three baseline OCR measurements in an assay medium (DMEM containing 10 mM glucose, 4 mM glutamine at pH 7.4 without bicarbonate), Oligomycin (1.0 μM), FCCP (0.125 μM), and antimycin A (10 μM) were injected sequentially with OCR and ECAR measurements recorded after each injection. The following mitochondrial respiratory functional characteristics were elucidated from the OCR trace: basal OCR (OCR in the absence of any mitochondrial inhibitors), ATP-dependent OCR (OCR necessary to synthesize ATP), reserve capacity (difference between the maximal and basal OCR), which is an estimate of the potential bioenergetic reserve the cell can call upon in times of stress, non-mitochondrial (OCR after the addition of Antimycin A) and proton leak. Glucose –, glutamine –, and FA – dependent MiST were performed using an assay medium only containing glucose (10 mM), glutamine (4 mM) or palmitate-BSA (0.17 mM) as substrate for energy production.

Basal=OCR without inhibitors−Antimycin inhibted OCR Eq1

Maximal=OCR due to FCCP−Antimycin inhibted OCR Eq2

Reserve Capacity=Maximal−Basal Eq3

Proton Leak=Oligomyin Inhibited OCR−Antimycin inhibted OCR Eq4

Non−mitochondrial=Antimycin inhibted OCR Eq5

#### GlyST

Three initial measurements were done in glucose free assay media (glutamine alone), which is followed by injection of glucose (10 mM), oligomycin (1.0 μM) and 2DG (100 mM) were added successively and OCR and ECAR were measured. Glycolytic parameters such as glycolytic rate (ECAR in the presence of glucose), glycolytic capacity (maximum attainable glycolysis), and glycolytic reserve (difference between glycolytic capacity and glycolytic reserve) were determined from the ECAR trace [50].

#### Mitochondrial oncobioenergetic index (MOBI)

To calculate MOBI, we used an equation as described before [11] with modification. The values were calculated from OCR trace generated using standard assay medium.

$$\text{MBI}=\text{Basal OCR}\times \log[\frac{\text{Reserve capacity}+\text{ATP Dependent}}{\text{Proton Leak}+\text{Non-mito}.\text{OCR}}]$$ Eq6

#### Measurement of endogenous and exogenous FAO

RWPE-1 and its tumorigenic clones were seeded at a density of 3 × 10^4^ cells/well in 96-well Seahorse cell culture microplates containing 80 μL of growth medium and incubated overnight at 37°C, in 5% CO_2_. Twenty four hours prior to the assay, the growth medium was replaced with 80 μL of substrate limited DMEM medium (0.5 mM glucose, 1 mM Glutamine, 0.5 mM carnitine, and 1% FBS). On the day of the assay, substrate limited medium was replaced with 135 μL of FAO assay medium [Krebs-Henseleit Buffer (KHB): 111 mM NaCl, 4.7 mM KCl, 1.25 mM CaCl_2_, 2 mM MgSO_4_, 1.2 mM NaH_2_PO_4_ supplemented with 2.5 mM glucose, 0.5 mM carnitine, and 5 mM HEPES, adjusted to pH 7.4 at 37°C] and incubated in a non-CO_2_ incubator for 45 minutes at 37°C. To determine the OCR contributed by FAO, wells were treated with either vehicle or 40 μM etomoxir (15 μL of 400 μM stock solution/well). Appropriate wells were then treated with 30 μL of BSA or Palmitate-BSA FAO Substrate (Seahorse Biosciences Cat# 102720-100) and immediately placed the microplate in the XF96 Analyzer and run the MiST.

OCR trace due to FAO is plotted to calculate individual bioenergetic parameters as described above.

OCR due to FAO=OCR without Eto−OCR with Eto Eq7

Endogenous and exogenous FAO is calculated from the following equation:

Endogenous FAO=OCR BSA without Eto−OCR BSA with Eto Eq8

Exogenous FAO=OCR palmitate BSA without Eto−OCR palmitate BSA with Eto Eq9

Various bioenergetic parameters were calculated from the OCR trace due to FAO as described above for MiST.

### Measurement of mitochondrial content

To determine the mitochondrial content in prostrate epithelial cells citrate synthase activity was measured, which is a reliable measure of cellular mitochondrial content as described before [22]. Citrate synthase activity in total lysate was determined as the rate of color change of 5,5′-dithiobis-(2-nitrobenzoic) acid at 412 nm. The reaction was initiated by the addition of oxaloacetate in the presence of Acetyl-CoA. The cells were lysed in tris buffer (200 mM, pH 8.0) containing 0.1% triton X-100 and protease inhibitor cocktail without EDTA [51]. The lysate is centrifuged and the supernatant (10 μL) was used for the analysis. The activity is normalized to the amount of protein in each sample.

Abundance of citrate synthase protein and VDAC/Porin in the same cell lysate was determined by western blot (10 μg/lane) using anti-citrate synthetase and anti-VDAC/Porin antibody (Abcam Cat # ab96600 and ab14734; 1:2000 and 1:2500 dilution respectively in 5% milk prepared in TBST; incubated overnight at 4°C) and secondary anti-rabbit IgG and anti-mouse IgG (1:6000 dilution prepared in 5% milk in TBST; 1.0 hr. incubation at room temperature). The blots were developed using ECL technique as described by the manufacturer (GE Life Sciences). The blots were imaged and semi-quantified using ImageJ Software. Protein amount was quantified using DC protein assay (Bio-Rad, Irvine, CA) Equal gel loading of protein was determined by Ponceau Staining.

### Measurement of cellular ATP levels

Cells were plated in 96-well plates in triplicates at a density of 2 × 10^4^ cells/well in complete media as describe above and incubated overnight at 37°C, in 5% CO_2_. The ATP levels were measured using commercially available CellTiter-Glo® (Cat# G7572) chemi-luminescent detection kit from Promega (Madison, WI). Manufacturer's instructions were followed to quantify the cellular levels of ATP.

### Statistical analysis

Data are mean ± SEM. Multiple groups were compared using one-way ANOVA, followed by Tukey post-tests. A *p* value < 0.05 was considered significant.

## Abbreviations

4-HNE: 4-hydroxynonenal
ATP: Adenosine tri-phosphate
CPT1B: Carnitine palmitoyltransferase IB
ETO: Etomoxir
FAO: fatty acid oxidation
FBS: fetal bovine serum
FCCP: Carbonyl cyanide 4-(trifluoromethoxy)phenylhydrazone
GAPDH: Glyceraldehyde 3-phosphate dehydrogenase
GlyST: Glycolytic stress test
HPV: human papilloma virus
MOBI: mitochondrial oncobioenergetic index
MiST: mitochondrial Stress test
MNU: N-Methyl-N-nitrosourea
OCR: oxygen consumption rate
OXPHOS: oxidative phosphorylation
PSA: prostate specific antigen
ROS: Reactive oxygen species
